# Case Report: Testicular teratoma with malignant transformation to melanoma and concurrent metastatic carcinoma of undetermined primary origin in a red deer (*Cervus elaphus*)

**DOI:** 10.3389/fvets.2025.1699289

**Published:** 2025-11-07

**Authors:** Miguel Criado, Paloma Prieto, Juan José Palomares, Desiderio Lopezosa, David Zapico, Pedro Mendívil, Julio Benavides, M. Carmen Ferreras, José Espinosa

**Affiliations:** 1 Facultad de Veterinaria, Departamento de Sanidad Animal, Universidad de León, León, Spain; 2 Instituto de Ganadería de Montaña, CSIC-ULE, León, Spain; 3 Parque Natural Sierras de Cazorla, Segura y Las Villas, Jaén, Spain

**Keywords:** teratoma, testis, malignant transformation, melanoma, carcinoma, *Cervus elaphus*

## Abstract

Testicular teratomas are rare germ cell neoplasms composed of two or more embryonic germ layers—ectoderm, mesoderm, and endoderm. They are uncommon in domestic species, with only a few cases reported in wild mammals, and malignant transformation is rare. We report the case of a European red deer (*Cervus elaphus*), approximately 4 years of age, that presented with marked scrotal enlargement. Necropsy revealed an enlarged left testis weighing 3.29 kg. On sectioning, it showed complete loss of its normal architecture, with multiple multinodular solid areas and cystic regions containing dark fluid. An inguinal lymph node was diffusely darkly pigmented, while an iliac lymph node was enlarged and showed complete architectural effacement. Histopathological examination confirmed the presence of a teratoma with differentiation into tissues derived from all three germ layers. Additionally, two distinct cell populations with clear malignant features were identified in the testis and the iliac lymph node. Immunohistochemical and immunofluorescence studies were performed for their characterization. Antibodies used included pancytokeratin (PCK), Melan-A, vimentin (Vim), c-KIT receptor tyrosine kinase (CD117), S100 protein, and ionized calcium-binding adaptor molecule 1 (IBA1), some of which had not previously been used in this species. The results revealed a malignant transformation of the teratoma, with the presence of a poorly differentiated, invasive melanoma (Vim^+^, Melan-A^+^, S100^−^, and CD117^−^), accompanied by numerous melanophages (IBA1^+^) in the surrounding tissue and inguinal lymph nodes, and a concomitant metastatic carcinoma (PCK^+^), which was identified in the iliac lymph node of undetermined origin. To the best of our knowledge, there are no documented cases of testicular teratomas with these characteristics in wild species of the Cervidae family or in other animal species.

## 1 Background

Growing interest in wildlife has led to an increasing number of reports on pathologies affecting these species. Nevertheless, information remains scarce in comparison, particularly concerning neoplastic and other non-infectious conditions. In domestic ruminants, testicular neoplasms are uncommon. Bulls are most frequently affected by interstitial cell tumors, whereas rams and bucks are rarely affected; however, occasional cases of seminomas and Sertoli cell tumors have been reported (1, 2). Although testicular teratomas are periodically described in young horses, particularly in cryptorchid testicles, these neoplasms are rare in domestic species. A systematic multi-database literature search conducted in PubMed and Google Scholar databases from the period 1900 to 2025 using combinations of keywords such as *testicular*, *testis*, *teratoma*, *tumor*, and *neoplasia* and species-related terms such as *Cervus elaphus*, *ruminant*, *cattle*, *bull*, *bovine*, *sheep*, *ram*, *ovine*, *goat*, *buck*, *caprine*, *red deer*, *cervid*, *Cervidae*, and *Bovidae* revealed no reports of testicular teratomas in domestic ruminants.

Similarly, in wild ruminants, testicular neoplasms are rarely documented; in cervids, only one seminoma (3) and one benign testicular teratoma (4) have been reported, both in captive gray brocket deer (*Mazama gouazoubira*). Only one other report on benign testicular teratoma was found in a wild ruminant, an immature nyala (*Tragelaphus angasii*) (5). The scarcity of reports likely reflects the rarity of these neoplasms and the difficulties associated with obtaining and studying wildlife samples. Consequently, data on the prevalence, morphology, and biological behavior of testicular tumors in wild ruminants remain scarce. Documenting such cases contributes to our understanding of comparative oncology, provides insights into tumor biology across species, and informs wildlife management and veterinary care practices.

Overall, testicular teratomas are rare in all species, and malignant transformation is even more uncommon. In this study, we report a case of a testicular teratoma with somatic malignant transformation in a European red deer (*Cervus elaphus*), highlighting its pathological features and its relevance for wildlife health surveillance.

## 2 Case history

**Figure 1:**
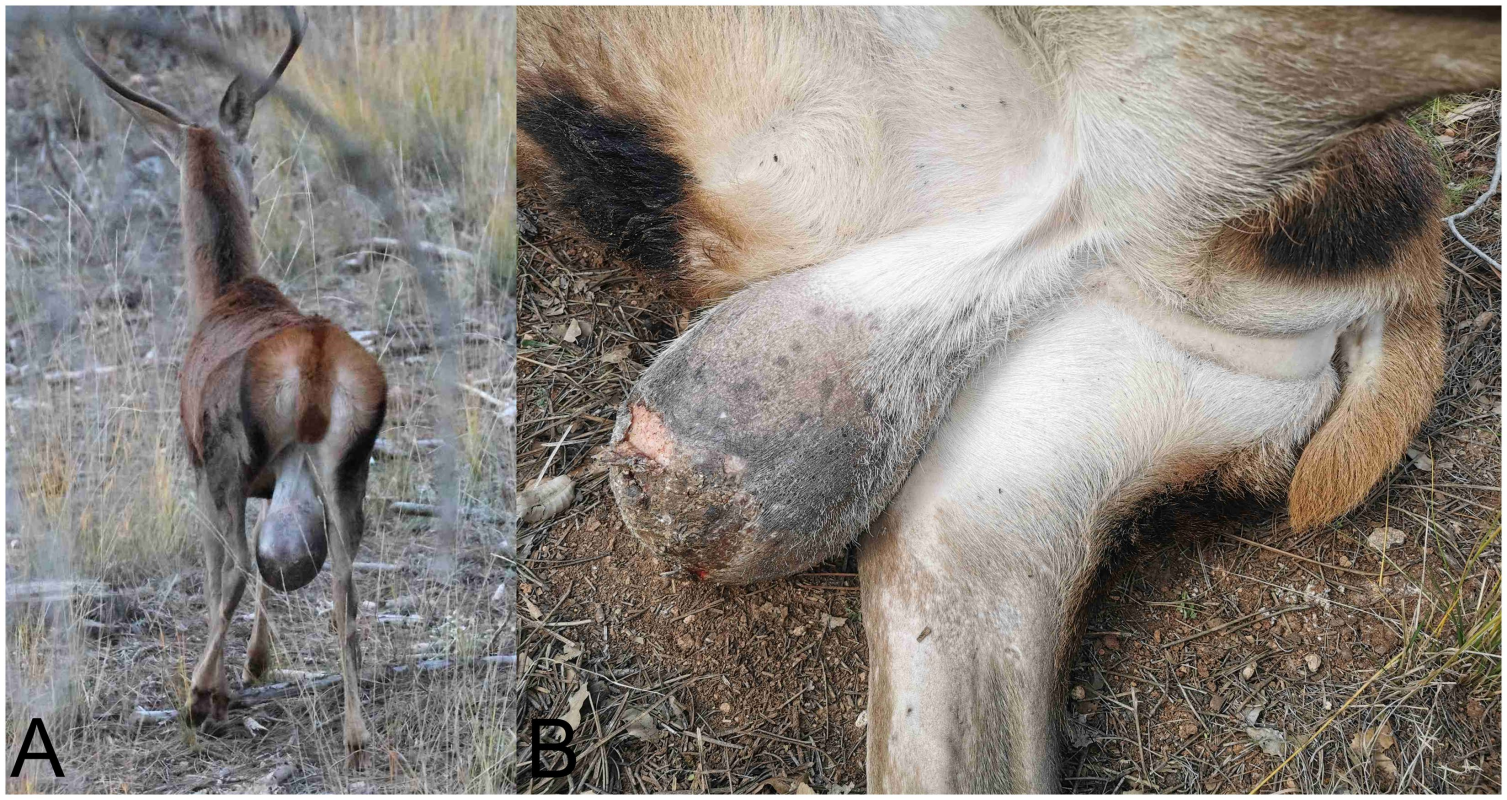
Scrotal enlargement in a red deer (*Cervus elaphus*). **(A)** Image of the specimen observed at a long distance in its natural habitat, showing a pronounced scrotal enlargement. **(B)** After culling, it was confirmed that this enlargement corresponded to a markedly enlarged testicular region, with areas of skin ulceration visible, possibly caused by the friction of the scrotal sac against surfaces.

A male European red deer (*Cervus elaphus*), approximately 4 years of age (fully erupted molars with moderate wear and antlers bearing three tines), was observed by environmental officers in the municipality of Santiago-Pontones (Jaén, Andalusia, Spain) exhibiting motor difficulties associated with a marked scrotal enlargement (Figure 1A). The animal showed an acceptable body condition for the species and season. However, as part of the population control program for this species within the Sierras de Cazorla, Segura y Las Villas Natural Park, given the size of the observed lesions and the locomotor impairment, the animal was humanely culled using a firearm. No additional therapeutic or supportive interventions were performed, consistent with standard wildlife management protocols.

## 3 Postmortem diagnosis

### 3.1 Necropsy and gross findings

**Figure 2:**
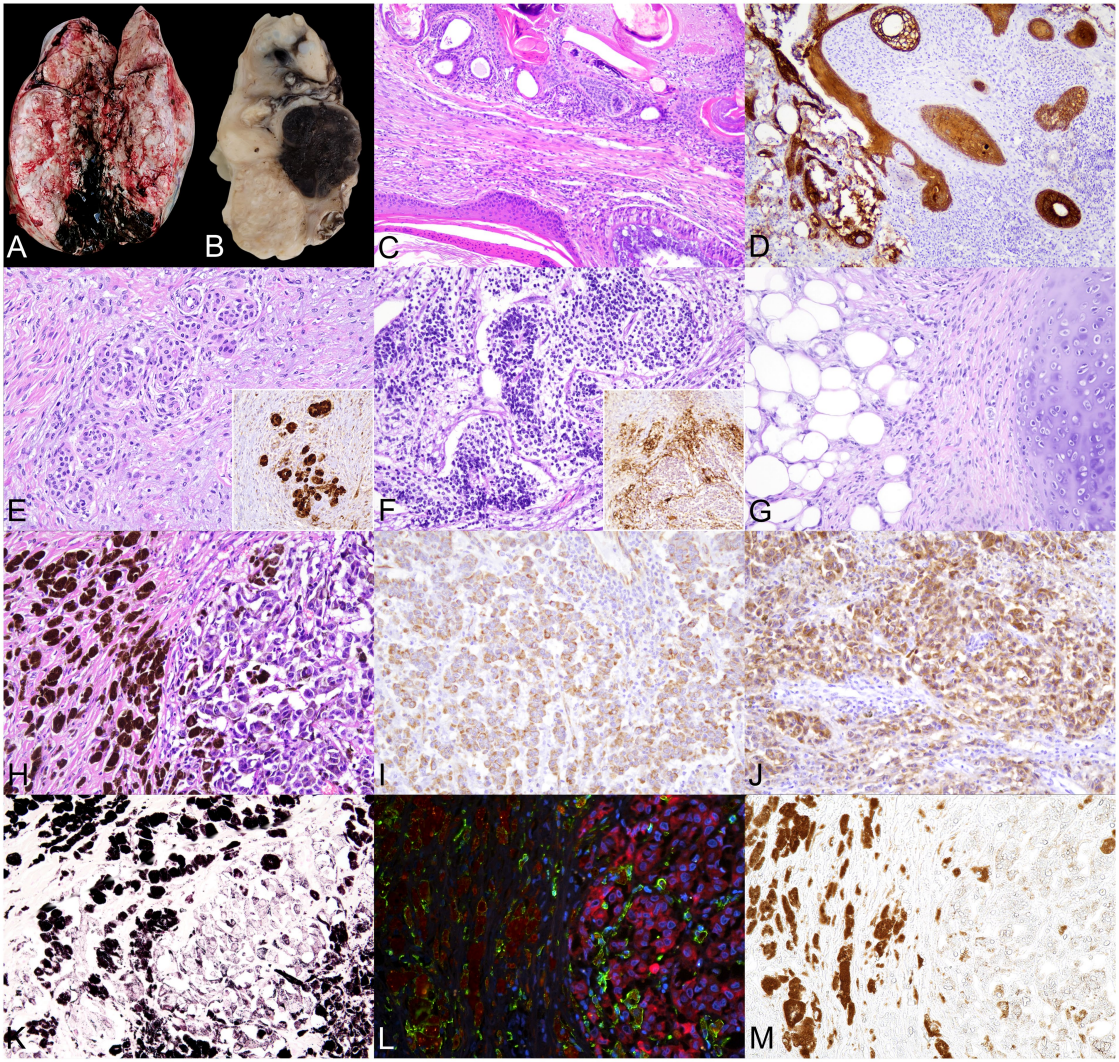
Right testis. Teratoma and hypomelanotic melanoma with extensive melanophage infiltration. **(A)** Gross images of the testicular parenchyma show complete loss of normal structure due to the presence of an irregular, poorly demarcated, multilobulated overgrowth. On sectioning, the tissue was firm, with a heterogeneous appearance, including alternating solid, necrotic, and cystic areas containing dark fluid. **(B)** Section of the fixed specimen shows, among other features (top to bottom), areas resembling cartilage and adipose tissue, multifocal cystic areas filled with amorphous material (keratin-like), and a well-defined nodular region of intense black color. **(C–G)** The teratoma displays **(C)** Multiple structures lined by ectodermal-derived epithelia (simple cuboidal epithelium and stratified squamous keratinized epithelium, upper and lower left) and endodermal-derived epithelia (ciliated columnar epithelium with goblet cells, lower right), 100×. **(D)** Immunohistochemistry (IHC) for pancytokeratin (PCK) highlights the diversity of epithelial components within the mass, 100×. **(E)** Possible Schwann cell cords show strong S100 immunoreactivity, confirming neural crest origin (inset: IHC for S100 of a similar area). **(F)** Immature neuroectodermal rosette-like formations are composed of small, densely packed, undifferentiated cells, surrounded by glial cells strongly positive for S100 and neuropil-like material (inset: IHC for S100 of a similar area). **(G)** Adipose and cartilaginous tissues of mesodermal origin are also present. **(H–K)** The invasive, poorly differentiated melanoma shows **(H)** Highly pleomorphic neoplastic cells with little melanin pigment on the right and numerous melanin-laden cells on the left. **(I)** IHC for vimentin demonstrates mesenchymal characteristics of these cells, **(J)** While IHC for Melan A confirms melanocytic differentiation. **(K)** Fontana–Masson staining confirms that the pigment in both cell types is melanin. **(L)** Immunofluorescence for ionized calcium-binding adapter molecule 1 (IBA1, green) and Melan A (red), with nuclei stained with DAPI (blue), reveals that melanin-laden cells are macrophages—melanophages (IBA1^+^)—whereas Melan A^+^ melanocytes contain little or no melanin; melanin autofluorescence appears brownish-orange. **(M)** Brightfield micrograph of the same slide and area shown in **(L)**, no additional staining was applied, reveals melanin presence. All slides were stained with H&E, and images were captured at 200 × magnification, unless otherwise noted.

**Figure 3:**
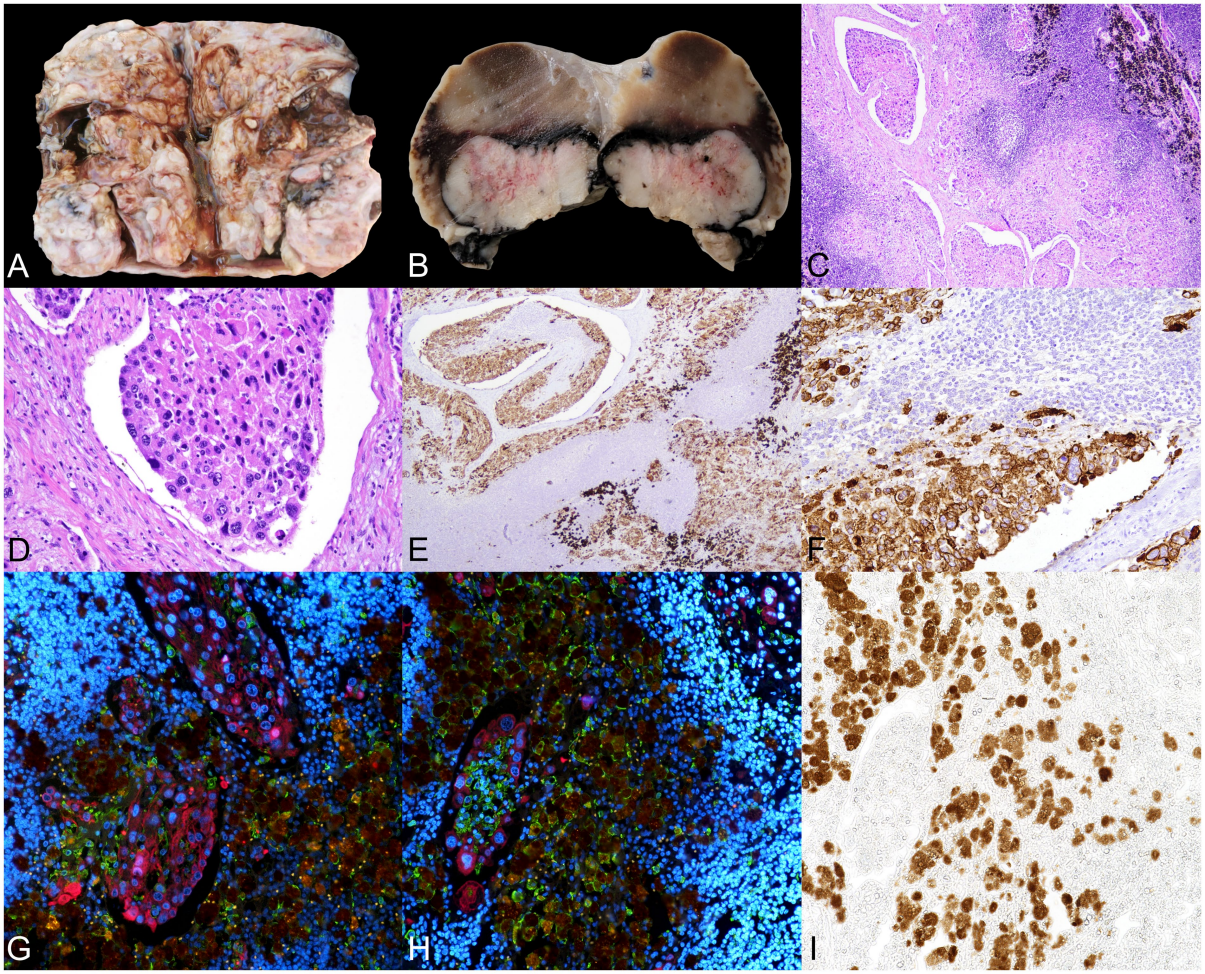
Mass observed in the intrapelvic region, compatible with a metastatic iliac lymph node. **(A)** Macroscopic image of the lymph node shows complete loss of normal tissue architecture, with an irregular nodular surface and cystic cavities filled with dark fluid. **(B)** Section of the fixed specimen shows more preserved lymphoid tissue adjacent to an irregular, firm nodular overgrowth, partially cleared and surrounded by a pigmented zone. **(C)** Microscopic images of the lesion reveal lymphoid tissue displaced by a pronounced desmoplastic reaction, along with multiple dilated lymphatic vessels filled with emboli of neoplastic cells exhibiting marked cellular atypia. Melanophages are also observed (upper left corner). H&E, 40×. **(D)** The neoplastic cells show anisokaryosis, with generally large, round nuclei and a moderate amount of cytoplasm. H&E. **(E)** Immunohistochemistry (IHC) for pancytokeratin (PCK) confirms the epithelial origin of the cells present within the vascular lumina, 100×. **(F)** Inset of E. Neoplastic emboli are frequently accompanied by mononuclear cells. **(G,H)** Immunofluorescence (IF) for ionized calcium-binding adapter molecule 1 (IBA1, green) and PCK (red), with nuclei stained with DAPI (blue). Melanin autofluorescence appears brownish-orange. **(G)** Several neoplastic emboli in a small area. **(H)** Monocytes and macrophages (IBA1^+^) constitute a significant component of the associated mononuclear inflammatory infiltrate. Melanin-laden cells correspond to melanophages. **(I)** Brightfield micrograph of the same slide and area shown in **(H)**; no additional staining was applied, which reveals melanin presence. All figures were captured at 200 × magnification, unless otherwise noted.

Field necropsy revealed that the scrotal enlargement corresponded to a markedly enlarged left testis (Figure 1B) measuring 25 × 17 × 5.5 cm and weighing 3.29 kg. On gross section, the testis showed complete loss of normal architecture, with the parenchyma replaced by multiple solid, multinodular areas interspersed with extensive regions of friable, white material with a dry texture and variably sized cystic spaces containing abundant dark fluid. The tunica albuginea was grossly intact, and no evidence of capsular penetration or invasion into adjacent tissues was observed (Figures 2A,B). The right testis was markedly reduced in size (8.2 × 7.3 × 3.4 cm), consistent with atrophy, and lacked gross lesions. The inguinal lymph nodes were diffusely enlarged but maintained their gross architecture and exhibited diffuse, dark brown to black discoloration (Supplementary Figure 1A). Within the abdominal cavity, adjacent to the iliac region, there was a discrete mass measuring 15.7 × 14.6 × 7.8 cm and weighing 0.89 kg. On section, the mass had a gross appearance similar to that of the affected left testis, comprising multifocal to coalescing necrotic areas and multiple cystic cavities filled with dark fluid (Figures 3A,B). Based on its anatomical location and gross features, the lesion was initially interpreted as an altered regional lymph node. No additional gross abnormalities were detected in other organs. Representative tissue samples from the described lesions and from major organs, such as the livers, kidneys, lungs, spleens, small and large intestines, heart, and skeletal muscle, were collected and referred to the Pathological Diagnostic Service, Faculty of Veterinary Sciences, University of León, Spain, for histopathological evaluation.

### 3.2 Histopathological, immunohistochemical, and immunofluorescence analyses

Tissue samples fixed in 10% buffered formalin were routinely processed through a graded alcohol series and xylene before being embedded in paraffin wax. Tissue sections of 3-μm thickness were obtained from each sample and stained with Harris’s hematoxylin and eosin (H&E). Additional histochemical stains, including Masson–Fontana, were also performed. Immunohistochemical (IHC) and immunofluorescence (IF) analyses were performed to characterize the cell populations present in the different lesions, employing antibodies against pancytokeratin (PCK), Melan-A, vimentin (Vim), receptor tyrosine kinase c-KIT (CD117), S100 protein, and ionized calcium-binding adapter molecule 1 (IBA1).

The IHC and IF staining were performed on 3 μm-thick tissue sections placed on poly-L-lysine-coated slides (SuperFrost Plus Adhesion slides—Thermo Fisher Scientific, Waltham, USA). After deparaffinization and hydration, the sections were washed twice in wash buffer (Agilent Technologies, Santa Clara, USA) for 5 min. Then, to block endogenous peroxidase or to reduce background fluorescence, the sections were immersed in 3% H_2_O_2_ in methanol for 30 min in darkness at room temperature, washed again, and, in some cases, antigen retrieval was performed using heat-based methods, as stated in Supplementary Table 1. After washing twice, the sections were incubated overnight at 4 °C in a humidified chamber with the primary antibodies (see Supplementary Table 1). Appropriate species and isotype-matched immunoglobulins were included as negative controls, while tissue samples from the same animal and from other species known to express the antigens of interest served as positive controls. Animal-Free Blocker® and Diluent R. T. U. (Vector Laboratories, CA, USA) were used for all antibody dilutions.

For IHC, after washing, the sections were incubated for 40 min at room temperature with the appropriate monoclonal or polyclonal antibody and horseradish peroxidase-labeled polymer (Agilent Technologies, Santa Clara, USA) and, after washing, antibody localization was determined using 3,3-diaminobenzidine (DAB, Agilent Technologies, Santa Clara, USA) as a chromogenic substrate for peroxidase. The sections were counterstained with Mayer’s hematoxylin.

For IF, after three washes, the sections were incubated for 1 h at room temperature with both secondary antibodies (see Supplementary Table 1). The Vector® TrueVIEW® Autofluorescence Quenching Kit (Vector Laboratories, CA, USA) was used to reduce tissue autofluorescence, and, after two washes, the slides were stained using 4′,6-diamidino-2-phenylindol (DAPI) (Invitrogen™, Carlsbad, CA, USA) at a concentration of 2.5 μg/mL before mounting. Micrographs were taken using the direct microscope Eclipse Ni-E (Nikon, Tokyo, Japan) and either the Prime BSI Scientific CMOS monochrome scientific camera (Photometrics® Prime BSI™, Scottsdale, AZ, USA) or the DS-Ri2 color microscope camera (Nikon, Tokyo, Japan).

### 3.3 Histopathological findings

Sections of the altered testis revealed the presence of tissue components derived from all three embryonic germ layers: ectoderm, mesoderm, and endoderm. Ectodermal components included various types of epithelia, such as keratinized stratified squamous, non-keratinized stratified squamous, and simple cuboidal. These were observed forming glandular structures resembling those present in the dermis (apocrine and sebaceous glands), as well as large cystic cavities filled with lamellar keratin, resembling cystic hair follicles (Figures 2C,D). An epithelial origin was confirmed by IHC studies, which showed strong positivity for PCK in all epithelial structures (Figure 2D). Additionally, cords of Schwann cells exhibiting immunopositivity for S100 and vimentin were identified (Figure 2E). Furthermore, areas compatible with immature neuroectoderm, characterized by rosette-like formations with peripheral glial-like (S100+, Vim+) cells and central S100^−^, Vim^+^ cells, were observed (Figure 2F). Mesodermal differentiation was represented by areas of hyaline cartilage composed of well-differentiated chondrocytes (S100^+^) embedded in a basophilic extracellular matrix, as well as the adipose tissue (Figure 2G). Endodermal differentiation was also evident, with large cystic structures lined by pseudostratified ciliated epithelium with goblet cells, resembling respiratory epithelium (Figure 2C). No areas of testicular parenchyma with preserved histological architecture were identified. Altogether, these findings were consistent with the presence of a testicular teratoma.

Additionally, 40% of the testicular mass was also comprised of an unencapsulated, poorly circumscribed, poorly demarcated, moderately cellular, and infiltrative neoplasm. It consisted of a pleomorphic cell population with spindle-to-oval morphology, scant and slightly basophilic cytoplasm, with a small proportion of cells (less than 20%) containing minor amounts of a granular, dark-brown pigment consistent with melanin. The nuclei were oval or elongate and centrally located. Moderate anisokaryosis and anisocytosis were observed, with occasional mitotic figures and instances of binucleation phenomena (Figure 2H). IHC studies showed positivity for vimentin (Figure 2I) and Melan-A (Figure 2J) and negativity for CD117 and S100, confirming the diagnosis of melanoma. Adjacent to the melanocytes, another cell population was frequently observed, composed of large cells with abundant cytoplasm filled with brown-dark pigment similar to that observed in the neoplastic cells, morphologically compatible with macrophages (Figure 2H). Masson–Fontana staining confirmed that the pigment in both cell types was melanin (Figure 2K). Since the DAB staining used in the IHC protocol interfered with the differentiation of melanin (Supplementary Figure 1B) and, consequently, with the identification of the pigment-containing cell types, IF was performed using IBA1 and Melan-A antibodies. IF revealed that the cells containing the largest amounts of melanin were melanophages, whereas the majority of melanocytes contained little to no melanin (Figures 2L,M). To evaluate the possibility of melanoma metastasis in the enlarged, pigmented inguinal lymph node, IHC for IBA1 and dual IF for IBA1 and Melan-A were performed. No neoplastic melanocytes were observed in this organ, and all pigment-laden cells were identified as macrophages (Supplementary Figures 1B–D).

A histological analysis of the mass observed in the caudal abdomen, located in the iliac region, was consistent with a lymph node whose structure was completely disrupted by the presence of an expansive, poorly demarcated, non-encapsulated neoplasm. It was composed of irregularly cuboidal to polygonal cells arranged in cords, trabeculae, and solid nests, supported by a moderately dense desmoplastic stroma. The neoplastic cells exhibited indistinct borders, moderate amounts of granular eosinophilic cytoplasm, and irregularly round to oval nuclei with finely stippled chromatin and one to two distinct nucleoli. Moderate anisokaryosis and anisocytosis, along with frequent mitotic activity, were observed. Multiple aggregates of neoplastic cells with similar characteristics were frequently observed within the lumina of local lymphatic vessels (Figures 3C,D), sometimes accompanied by mononuclear cell infiltrates. The neoplastic cells demonstrated strong immunopositivity for PCK (Figures 3E,F), confirming their epithelial origin. The inflammatory infiltrate surrounding the neoplastic cells consisted primarily of monocytes/macrophages (IBA1^+^) (Figures 3G,H), with occasional melanophages interspersed among the neoplastic population or residual lymphoid tissue (Figures 3C,G–I). These results confirmed the presence of a malignant epithelial tumor (carcinoma) of undetermined origin, in the absence of a similar population being detected within the teratoma.

To summarize, the results confirm somatic-type malignant transformation of a testicular teratoma to melanoma based on the detection of a poorly differentiated, hypomelanotic, invasive melanoma (Vim^+^, MelanA^+^, S100^−^, and CD117^−^), accompanied by numerous melanophages (IBA1^+^) in the surrounding tissue and inguinal lymph nodes. A metastatic carcinoma (PCK^+^) was also detected within an iliac lymph node; however, its primary site remains unidentified.

## 4 Discussion

Teratomas are germ cell tumors composed of tissues derived from two or more embryonic germ layers. Although they can occur in extragonadal locations, they most commonly develop in gonadal sites, particularly the ovaries and testes. The precise mechanisms underlying their development remain unclear, but they are thought to result from errors in germ cell migration during embryogenesis (6, 7). As previously noted, with the exception of young horses, testicular teratomas are very rarely reported in both domestic and wild animals. A systematic literature search revealed only two reports of testicular teratomas in ruminants (4, 5) and, in both cases, no malignant components were identified. Consequently, the origins, development, and prognosis of these tumors remain poorly characterized in non-human species. In humans, prepubertal teratomas generally have a better prognosis than postpubertal cases (7), but the absence of a clinical history in the case presented here makes it impossible to determine its origin or developmental timeline.

The histopathological features of this tumor—including the disorganized arrangement of mature and immature tissues from all germ layers, the presence of areas compatible with immature neuroectoderm (8), and findings suggestive of malignant transformation—support its classification as an immature teratoma with a poor prognosis. Malignant somatic transformation of a teratoma is rare; in humans, it occurs in only 3–6% of cases (9). A retrospective review of 24 cases revealed that 50% were adenocarcinomas and 50% were sarcomas (10), with only a few reports describing a malignant component attributable to melanoma. The majority of documented cases involve ovarian teratomas, with the most recent review estimating a prevalence of 0.2–0.8% and identifying 47 relevant reports (11–15), and only one melanotic neuroectodermal tumor has been reported, as a predominant component of an immature testicular teratoma in a 17-year-old man (16). Given the rarity of melanocytic differentiation within teratomas, immunohistochemical confirmation is critical; accordingly, in humans, over 95% of melanomas are S100^+^, with only a small percentage of primarily metastatic melanomas being S100^−^ (17, 18). In dogs and cats, retrospective studies indicate lower percentages of S100 antigenicity of 75–90% (19–21). Apparent loss of S100 expression can result from technical factors, anatomic location (17), or species-specific variability. In the present case, strong S100 positivity was observed in neural tissue within the same sections where the melanoma component was clearly S100^−^. This makes a technical artifact unlikely and supports the fact that the melanoma is genuinely S100^−^, likely reflecting aberrant differentiation of the melanocytic component arising within the teratoma, whereas the neoplastic melanocytes still produced variable amounts of melanin and expressed Melan-A. Additionally, a systematic literature search using the terms *teratoma*, *melanoma*, *melanocytic*, and names of common domestic species identified no previous reports of malignant transformation of a teratoma—testicular or otherwise—to melanoma. This case may therefore represent the first report of melanocytic transformation of a teratoma in veterinary medicine.

The presence of a metastatic carcinoma in an iliac lymph node raises the possibility of dual malignant transformation in this case; the absence of lesions elsewhere and the large size of the primary tumor support this hypothesis; however, this finding cannot be confirmed due to the absence of a corresponding malignant epithelial population within the teratoma. While the teratoma cannot be entirely excluded as the primary site, this remains undetermined. This study also underscores a limitation of sampling in large, heterogeneous tumors, where focal malignant populations may be overlooked. Nevertheless, the metastasis confirms malignant disease of undetermined origin, which may correlate with a poorer prognosis. Although a few reports of teratomas exist in cervids (4, 22, 23) and other species (24–26), to the best of our knowledge, no previous cases of testicular teratomas exhibiting similar features have been documented in the family Cervidae or in other animal species. Reports of somatic malignant transformation of testicular teratomas into a single malignant cell type in non-human animals are also very limited (24, 27); thus, malignant transformation —possibly dual—could be considered an extremely rare event. These tumors are likely to be exceedingly rare in both wild and domestic animals. Furthermore, underreporting due to limited pathological investigations, particularly in wildlife, may contribute to their scarcity in the literature. Although this case is incidental and exceptionally rare, it highlights the importance of ongoing wildlife health surveillance programs, which enable the detection and documentation of unusual pathological conditions that would otherwise remain unnoticed. Additionally, diagnostic challenges associated with detecting multiple malignant components within a teratoma—requiring comprehensive histopathological and immunohistochemical analyses—may result in incomplete characterization or missed diagnoses in routine veterinary practice. Notably, some of the antibodies used in this study—to the best of our knowledge—had not been previously applied to this or closely related species, highlighting the novelty and diagnostic relevance of our findings.

## Data Availability

The original contributions presented in the study are included in the article/Supplementary material, further inquiries can be directed to the corresponding author/s.
