# Supplementary material for: Case Report: Testicular teratoma with malignant transformation to melanoma and concurrent metastatic carcinoma of undetermined primary origin in a red deer (Cervus elaphus)
Source: Front Vet Sci. 2025 Nov 7;12:1699289. doi: 10.3389/fvets.2025.1699289 (PMC12635998; doi:10.3389/fvets.2025.1699289)

Supplementary Material

Supplementary Table 1. Antibodies, unmasking technique and dilutions used for immunohistochemistry and immunofluorescence (IF). Heat-induced epitope retrieval (HIER), deparaffination and hydration were performed using PT-link and Dako Target Retrieval Solutions at 95 °C for 20 minutes (Agilent Technologies). [a]For IF. [b]Secondary antibodies for IF.

Target	Clone/Reference	Epitope demasking	Dilution	Supplier
**S100b**	ab41548	HIER, pH 6.0	1:1000	Abcam (Cambridge,UK)
**Melan-A**	A103/ MA514168	HIER, pH 6.0	1:100	Invitrogen (Waltham, MA, US)
**Vimentin**	V9/M0725	HIER, pH 6.0	1:1000	Agilent Technologies (Santa Clara, CA, USA)
**Ionized calcium-binding molecule 1 (IBA1)**	019-19741	HIER, pH 6.0	1:1000	Wako (Japan)
**c-KIT (CD117)**	44-496G	HIER, pH 6.0	1:100	Invitrogen (Waltham, MA, USA)
**Pan-cytokeratin (PCK)**	MAD-211000Q	HIER, pH 9.0<br>HIER, pH 6.0[a]	1:100	Master Diagnostica (Granada, Spain)
**Goat Anti-Rabbit IgG H&L (Alexa Fluor 488)[b]**	ab150077		1:500	Abcam (Cambridge,UK)
**Goat anti-Mouse IgG (H+L) Cross-Adsorbed Secondary Antibody, Alexa Fluor 647 [b]**	A-21235		1:500	Invitrogen (Carlsbad, CA, USA)

Supplementary Figure 1. Inguinal lymph node. Diffuse melanin pigmentation. (a) Grossly, a diffuse black pigmentation could be observed. (b) Immunohistochemistry (IHC) for ionized calcium binding adaptor molecule 1 (IBA1), some macrophages are loaded with melanin (melanophages), but DAB labelling and melanin were difficult to discern. (c). Immunofluorescence for IBA1 (green) and Melan A (red); nuclei stained with DAPI (blue). Melanin autofluorescence appears brownish orange. Melanin-loaded cells were macrophages, and no melanocytes were present in the lymph node. (d) Brightfield micrograph of the same area shown in c, highlighting the melanin pigment.

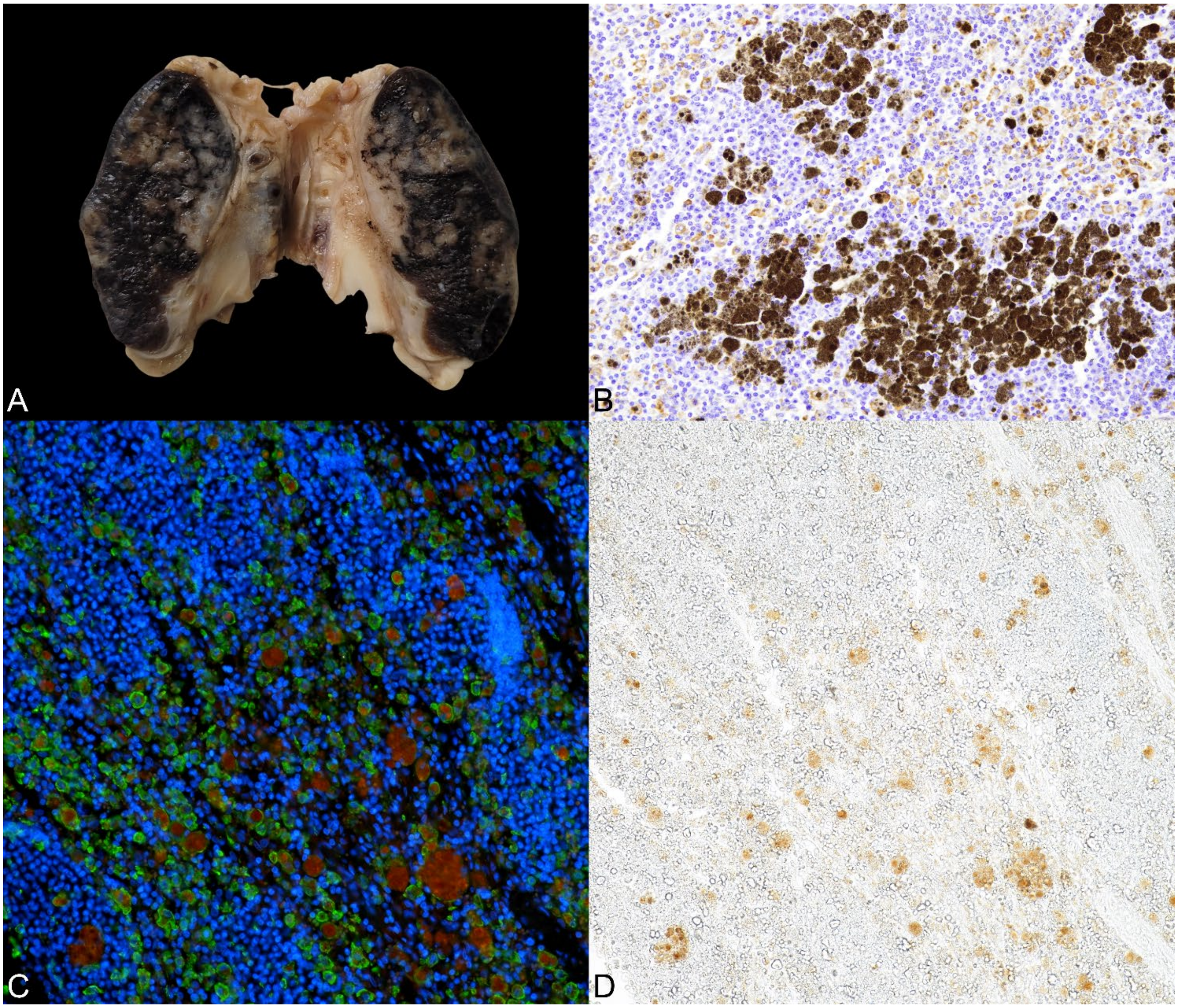

Supplement: Supplementary file 1 [file Data_Sheet_1.PDF]
